# Different acupuncture and moxibustion therapies for atopic dermatitis: a systematic review and network meta-analysis

**DOI:** 10.1186/s13643-026-03314-7

**Published:** 2026-09-01

**Authors:** Qianqian Liu, Zhujuan Wu, Xinyu Wang, Ting Li, Yuefang Chen

**Affiliations:** 1 Hangzhou Third People’s Hospital, Hangzhou, China; 2 Nursing Department, Sir Run Run Shaw Hospital, Zhejiang University School of Medicine, Hangzhou, China

**Keywords:** Atopic dermatitis, Acupuncture and moxibustion therapy, Network meta-analysis

## Abstract

**Objective:**

To investigate the effectiveness of different acupuncture and moxibustion therapies in improving symptoms in patients with atopic dermatitis (AD) to determine the optimal approach. This study aims to provide insights into clinical decision-making in the complementary and alternative management of AD.

**Study design:**

Systematic review and Network meta-analysis.

**Materials and methods:**

Randomized controlled trial (RCT) studies were retrieved from various databases, including PubMed, Embase, the Cochrane Library, Web of Science, CNKI, WANFANG Data, and VIP. The RoB 2.0 assessment tool was used to assess the risk of bias in the included studies. The meta-analysis was conducted via Stata 17 software.

**Results:**

A total of 23 RCTs involving 1,658 participants were included. Compared with controls, traditional pairwise meta-analyses revealed that acupuncture and moxibustion therapies significantly improved the overall response rate (OR: 2.83, 95% CI: 2.05–3.89; I^2^ = 43.7%, P = 0.025) and reduced the SCORAD score (SMD: –0.24, 95% CI: –0.37–0.11; I^2^ = 0.0%, P = 0.784). Network meta-analysis further indicated that moxibustion, embedding needling, and fire needling showed a higher probability of clinical efficacy according to SUCRA rankings. However, considering the small sample sizes of certain trials and the reliance on indirect evidence, these results should be interpreted as preliminary evidence rather than definitive clinical superiority.

**Conclusions:**

Acupuncture and moxibustion therapies appear to be effective in improving clinical outcomes in patients with atopic dermatitis, with moxibustion showing the greatest potential benefit. These findings support the therapeutic value of acupuncture-based interventions as adjunctive treatments for AD. However, further high-quality, large-scale randomized controlled trials are needed to confirm these results and to optimize clinical application strategies.

**Systematic review registration:**

PROSPERO CRD42025632518.

**Supplementary Information:**

The online version contains supplementary material available at https://doi.org/10.1186/s13643-026-03314-7.

## Highlights

- We compared acupuncture and moxibustion therapies for atopic dermatitis.
- This study comprehensively included 10 types of acupuncture and moxibustion therapies.
- SCORAD and overall response rate were used as outcome indicators for network meta-analysis.
- Moxibustion ranked first and appeared to be the most effective acupuncture and moxibustion therapy for atopic dermatitis.

**Supplementary Information:**

The online version contains supplementary material available at https://doi.org/10.1186/s13643-026-03314-7.

## Introduction

Atopic dermatitis (AD) is a chronic relapsing and remitting inflammatory skin disease that frequently manifests as recurrent xerosis, chronic eczematous lesions, and intense pruritus [1]. The Global Burden of Disease Study estimated that the general prevalence of AD was 15% ~ 20% among children and 2% ~ 10% among adults, indicating that AD is the most burdensome nonfatal dermatological condition worldwide [2]. AD has been found to be associated with increased risks of psychological comorbidities, sleep disturbances, substantially impaired quality of life and increased healthcare expenditure [3–6]. Therefore, providing effective management for AD patients is imperative.

Treatment strategies for AD are diverse and can be broadly divided into pharmacological and nonpharmacological approaches. Pharmacological therapies, such as antihistamines, immunosuppressants, corticosteroids, and Janus kinase (JAK) inhibitors, offer certain clinical benefits [7]. However, long-term use may lead to drug dependence. High medical costs and adverse effects further compromise patient adherence, making sustained treatment challenging and increasing the likelihood of relapse after discontinuation [8]. As a result, interest in exploring nonpharmacological complementary and alternative therapies for AD is increasing.

Acupuncture and moxibustion (AM) therapy, as a widely utilized complementary and alternative medicine, has demonstrated promising clinical potential for its therapeutic effects and safety profile in managing various chronic conditions [9, 10]. AM is increasingly recognized as viable complementary and alternative medicine (CAM) options for AD. Clinical practice and several international guidelines, such as those from the American Academy of Dermatology, acknowledge the utilization of acupuncture by patients to alleviate pruritus and improve quality of life [11]. AM modalities, including manual acupuncture and acupressure, are proposed to modulate the neuro-endocrine-immune axis and suppress Th2-mediated allergic inflammation [12]. However, despite their clinical use, high-level evidence regarding their comparative efficacy remains fragmented, necessitating this network meta-analysis. The comparative effectiveness of these various acupuncture techniques remains unclear, and direct head-to-head evidence is limited. Most randomized controlled trials (RCTs) have employed standard care as the control and seldom directly compared different AM interventions. Traditional pairwise meta-analyses are restricted to evaluating two interventions at a time and therefore cannot provide a comprehensive assessment of the relative effectiveness of multiple AM approaches for AD. Network meta-analysis (NMA) offers a methodological advantage by enabling the simultaneous comparison of multiple interventions, even in the absence of direct comparisons. Furthermore, NMA can generate treatment rankings on the basis of various outcomes, thereby assisting healthcare professionals in making informed decisions regarding optimal treatment strategies. Accordingly, we conducted a meta-analysis incorporating an NMA to compare the efficacy of different AM modalities for the treatment of AD by synthesizing existing evidence. Specifically, our aim was to identify the most effective AM intervention to support clinical decision-making in the complementary and alternative management of AD.

## Materials and methods

This research protocol was registered in the PROSPERO public database (registration number CRD42025632518) and strictly adhered to the guidelines for network meta-analysis outlined in the Preferred Reporting Items for Systematic Reviews and Meta-Analyses (PRISMA) [13].

### Literature retrieval strategy

A computer-based search was conducted to retrieve RCTs on AM therapies for treating AD. Both English and Chinese databases were searched, including PubMed, Embase, the Cochrane Library, Web of Science, China National Knowledge Infrastructure (CNKI), WANFANG Data, and the China Science and Technology Journal Database (VIP). The comprehensive literature search was executed across all selected databases on January 10, 2025. To ensure maximum coverage, no publication year restrictions were applied, and each database was searched from its respective inception date through the final search date. The retrieval strategy was based on the combination of subject words and free words. To compare the efficacy of acupuncture therapies as comprehensively as possible, we included interventions that acted on acupoints and acupoint areas, including sham acupuncture needling, acupuncture, fire needles, electroacupuncture, puncture and cupping, moxibustion, acupoint stimulation, needle embedding, and flying needles. The specific search strategy is shown in Supplementary file. In addition, further manual searches of the references included in the study were conducted to obtain additional relevant publications.

### Inclusion and exclusion criteria

The inclusion and exclusion criteria were established according to the Population, Intervention, Comparison, Outcome (PICOS) framework. The inclusion criteria were as follows: (P) population, individuals with a definite diagnosis of AD, with no restrictions on sex, age, or race. (I) Intervention, acupuncture and moxibustion therapies. (C) Compared with the control group, the control group was defined as receiving placebo or conventional care, and the interventions used included basic treatment, health education, Western medicine, and standardized Chinese herbal medicine. (O) Outcome, overall response rate (ORR) and/or scores on the SCORing Atopic Dermatitis Index (SCORAD). A detailed summary of the ORR definitions and thresholds for each study is provided in Supplementary Table S1.

The exclusion criteria were as follows: studies without clear diagnostic criteria for AD and studies not designed as RCTs. Studies were excluded if they failed to report primary outcomes in a format usable for meta-analysis and if these data remained unavailable after at least two attempts to contact the original authors via email. Additionally, publications with irretrievable full-text documents or non-English/Chinese language sources were systematically excluded.

### Literature screening and data extraction

Two independent investigators (K = 0.83 for titles, abstracts, and full-texts, P < 0.001) screened the literature, extracted information and cross-checked it. Any disagreements were resolved through discussion or consultation with a third researcher. The extracted data included the first author, publication year, country, sample size (control and intervention groups), age, sex, intervention measures, and outcome indicators.

### Risk of bias assessment

Dual independent evaluation of methodological quality was conducted by two trained methodologists (K = 0.901, P < 0.001) following the Cochrane risk of bias 2.0 (RoB 2) tool [14]. (i) generation of random allocation sequences; (ii) concealed grouping; (iii) blinding of patients and physicians; (iv) blinding of the evaluation of outcomes. (v) Incomplete outcome data; (vi) selective reporting of outcomes; and (vii) other biases. Red indicates high risk, green indicates low risk, and yellow indicates unclear risk. Disagreements were resolved by consensus or, if necessary, through adjudication by a third reviewer.

### Statistical analysis

We conducted a frequentist network meta-analysis to compare the effects of different acupuncture and moxibustion (AM) therapies. Traditional pairwise meta-analysis was performed for direct comparisons. Heterogeneity was assessed via the I^2^ value, and if I^2^ was ≥ 50%, a random effects model was applied to estimate the effect size. Otherwise, a fixed-effects model was used for data synthesis to ensure the robustness of the analysis [15]. In our review, standardized mean differences (SMDs) were used to calculate effect sizes for continuous outcomes due to substantial heterogeneity in baseline characteristics and sample sizes across studies. For dichotomous outcomes, odds ratios (ORs) were employed as the summary measure [16]. In the evidence network diagram, the size of each node represents the sample size for each intervention, whereas the thickness of the connecting lines indicates the number of studies comparing the interventions. Global inconsistency was assessed using the design-by-treatment interaction model. For outcomes with closed loops, loop-specific inconsistency was evaluated using the back-calculation method to identify any local incongruity between direct and indirect evidence. Model fit was appraised by examining the residual deviance and the Akaike Information Criterion (AIC). For outcomes without closed loops, a consistency model was directly applied as per standard NMA protocols. For each outcome, the efficacy of interventions is ranked via the surface under the cumulative ranking curve (SUCRA). All analyses were conducted in Stata 17.

## Results

### Results of literature retrieval

**Fig. 1:**
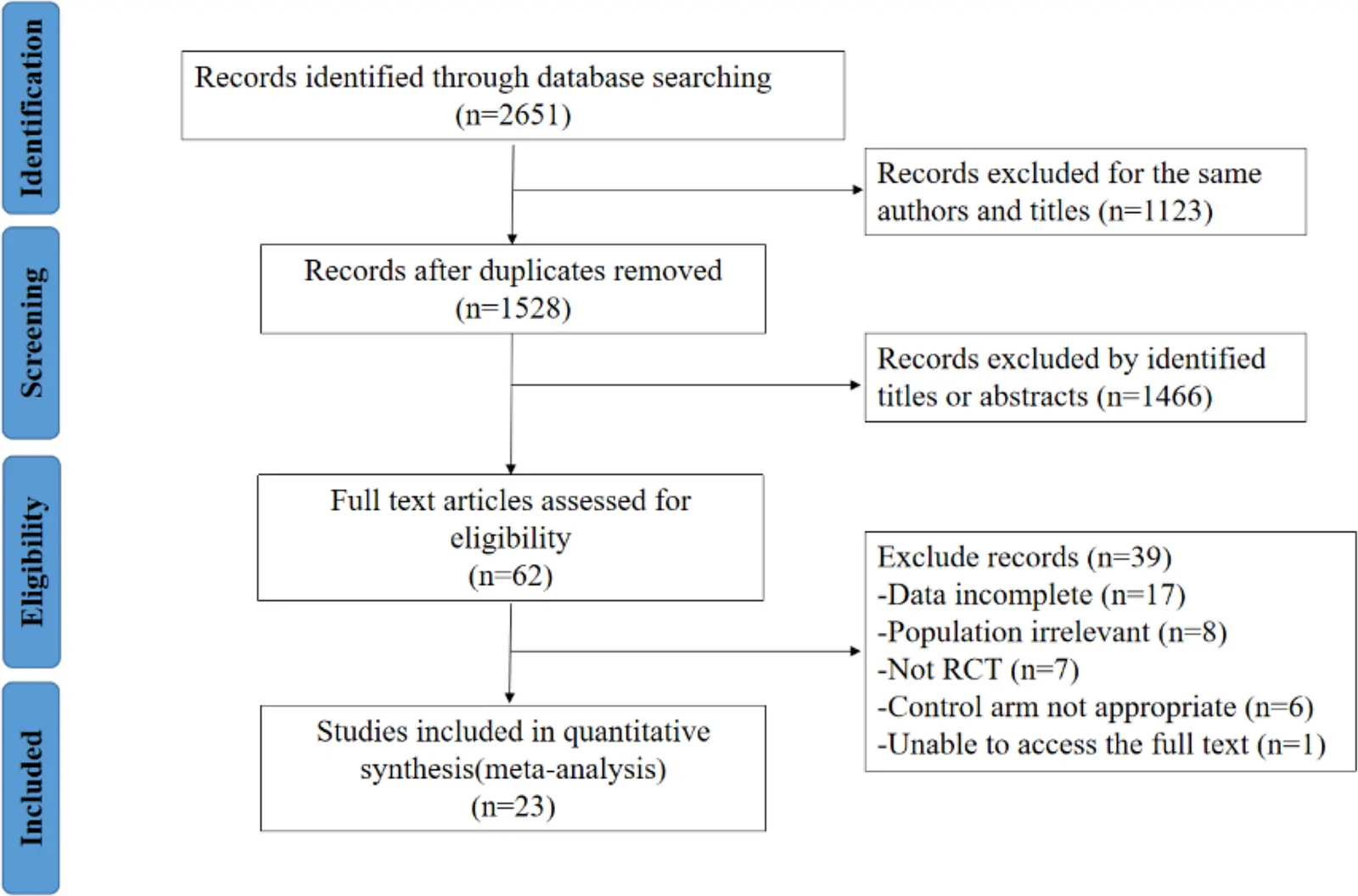
Flow chart of literature screening

A total of 2651 articles were initially identified, out of which 1123 duplicate articles were excluded. Following review by title and abstract, an additional 3,384 articles were excluded. Following a full-text review, 1466 articles were further excluded. Sixty-two articles progressed to full manuscript review. Of these, 39 were excluded because they did not fulfil our inclusion criteria. The remaining 23 studies [17–39] were included in this review. The detailed process is illustrated in Fig. 1.

### Basic characteristics of the included studies

Table 1 systematically delineates the general characteristics of the included studies, including their methodological parameters, geographical distributions, and temporal trends in accordance with the PRISMA 2020 reporting guidelines. The evidence synthesis encompassed 23 clinical trials (N = 1,658 participants; mean sample size = 72.1 ± 40.1), with geographical stratification revealing predominant recruitment in East Asia: 17 trials (73.9%) originated from China, and 3 trials (13.0%) originated from South Korea. Single-center designs were identified in the United States, Russia, and Germany, collectively accounting for 10.6% (177/1658) of the pooled population. Among the identified trials, two were three-arm studies. However, considering the type of exercise and study population, we selected data from only one intervention group and the control group in three-arm studies. The temporal distribution of the included studies spanned three decades from 1995–2024. The intervention measures in the control group included usual care, health education, placebo, sham acupuncture and Western medicine. In the intervention group, 8 therapies were used: acupuncture (ACU), fire needle (FIN), electroacupuncture (ELEC), puncture and cupping (PUNC), moxibustion (MOX), acupoint application (ACUP), embedding needle (IMN) and flying needle (FLN).

**Table 1:** Characteristics of the included studies

Author	Year	Region	Sample Size (CG/EG)	Sex(Male/Female)	Age (CG, EG Mean ± SD)	Intervention	Outcomes
Wu^17^	2024	China	50/50	CG 24/26 EG 21/29	45.04 ± 2.48, 44.96 ± 2.52	CG: western medicine EG: acupuncture	F1; F3
Li^19^	2023	China	30/30	CG 14/16 EG 17/13	38.23 ± 11.11, 36.10 ± 11.46	CG: usual care EG: electroacupuncture	F1; F2
Chen^20^	2023	China	57/57	CG 30/27 EG 32/25	41 ± 4, 40 ± 4	CG: western medicine EG: fire needle	F1; F3
Rotter^18^	2023	Germany	42/39	CG 11/31 EG 11/28	31.1 ± 10.8, 31.4 ± 10.9	CG: usual care EG: acupuncture	F2
Yao^21^	2023	China	51/51	CG 26/25 EG 23/28	25.68 ± 3.33, 25.46 ± 3.15	CG: usual care EG: acupuncture	F1; F2
Wang^22^	2022	China	30/30	CG 16/14 EG 17/13	35.21 ± 3.72, 34.95 ± 3.67	CG: western medicine EG: fire needle	F1; F2
Wang^23^	2021	China	30/30	CG 15/15 EG 16/14	Not reported	CG: western medicine EG: fire needle	F1; F2
Liu^25^	2021	China	33/33	CG 18/15 EG 16/17	33 ± 9, 34 ± 10	CG: western medicine EG: acupuncture	F1; F2
Jiang^26^	2021	China	27/27	CG 13/14 EG 12/15	6, 8	CG: acupuncture EG: moxibustion	F1
Park^24^	2021	Korea	17/18	CG 7/10 EG 7/11	22.82 ± 4.71, 24.67 ± 5.27	CG: placebo EG: acupuncture	F2
Lee^27^	2019	Korea	7/7	CG 1/6 EG 1/6	34 ± 5.68, 30.71 ± 7.57	CG: acupuncture EG: embedding needle	F2
YangY^29^	2018	China	30/30	CG 17/13 EG 14/16	11 ± 4, 13 ± 4	CG: acupuncture EG: embedding needle	F1; F2; F3
YangQ^28^	2018	China	60/60	CG 19/31 EG 27/33	23.37 ± 4.05, 24.52 ± 4.17	CG: western medicine EG: acupuncture	F1; F2; F3
Li^30^	2018	China	42/42	CG 28/14 EG 29/13	7.21 ± 2.6, 7.24 ± 2.25	CG: western medicine EG: embedding needle	F1; F3
Kang^31^	2018	Korea	10/10	CG 4/6 EG 4/6	23.9 ± 5.6, 22.5 ± 5.72	CG: sham acupuncture EG: acupuncture	F2
Chen^32^	2016	China	33/66	CG 15/18 EG 17/39	20.12 ± 7.15, 21.42 ± 6.96	CG: usual care EG: acupuncture	F1; F2; F3
Meng^33^	2015	China	12/20	CG 4/6 EG 11/11	55.7 ± 5.2, 54.4 ± 6.3	CG: western medicine EG: electroacupuncture	F1; F3
Quan^34^	2014	China	30/29	CG 14/16 EG 13/16	23.45 ± 8.12, 24.30 ± 9.18	CG: usual care EG: flying needle	F1; F2
Fu^36^	2012	China	43/43	CG 20/23 EG 18/25	Not reported	CG: western medicine EG: puncture and cupping	F1; F2; F3
Lee^35^	2012	United States	7/8	CG 6/1 EG 4/4	36, 34	CG: usual care EG: acupuncture	F3
Sun^37^	2009	China	70/130	CG 37/33 EG 65/65	1.2 ± 0.5, 1.2 ± 0.7	CG: western medicine EG:acupoint application	F1; F3
Adaskevich^38^	2000	Russia	31/50	Not reported	Not reported	CG: usual care, EG: acupuncture	F1
Xia^39^	1995	China	21/35	CG 12/9 EG 21/14	Not reported	CG: western medicine, EG: acupuncture	F1

CG, control group; EG, experimental group; F1, response rate; F2, SCORAD; F3, adverse effect

### Risk of bias

**Fig. 2:**
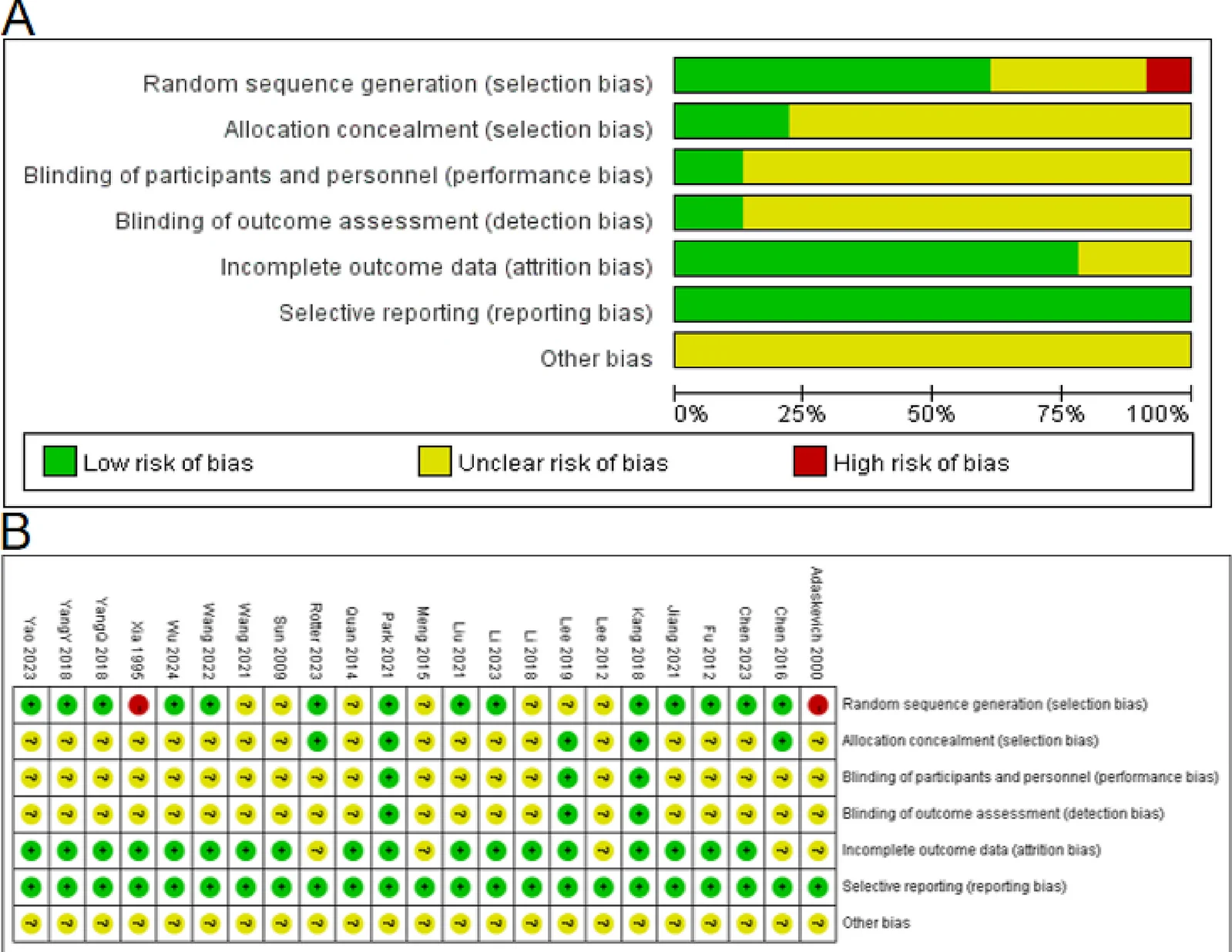
Risk of bias graph

Two researchers independently evaluated the risk of bias (RoB 2.0) according to the revised Cochrane risk of bias tool for the purposes of randomized trials. In accordance with the Cochrane criteria, the overall risk of bias in the included studies is shown in Fig. 2. The lack of a detailed description of random sequence generation and blinding resulted in uncertain risks. Combined with the GRADE guidelines, the quality of the included trials was moderate.

### Traditional pairwise meta-analysis and network meta-analysis

The scoring atopic dermatitis (SCORAD) index, a widely accepted tool for the screening and assessment of AD patients [40, 41], was selected as the primary outcome measure, whereas the overall response rate (ORR), a commonly used clinical endpoint in trials, was employed as a key supplementary outcome.

#### Overall response rate

**Fig. 3:**
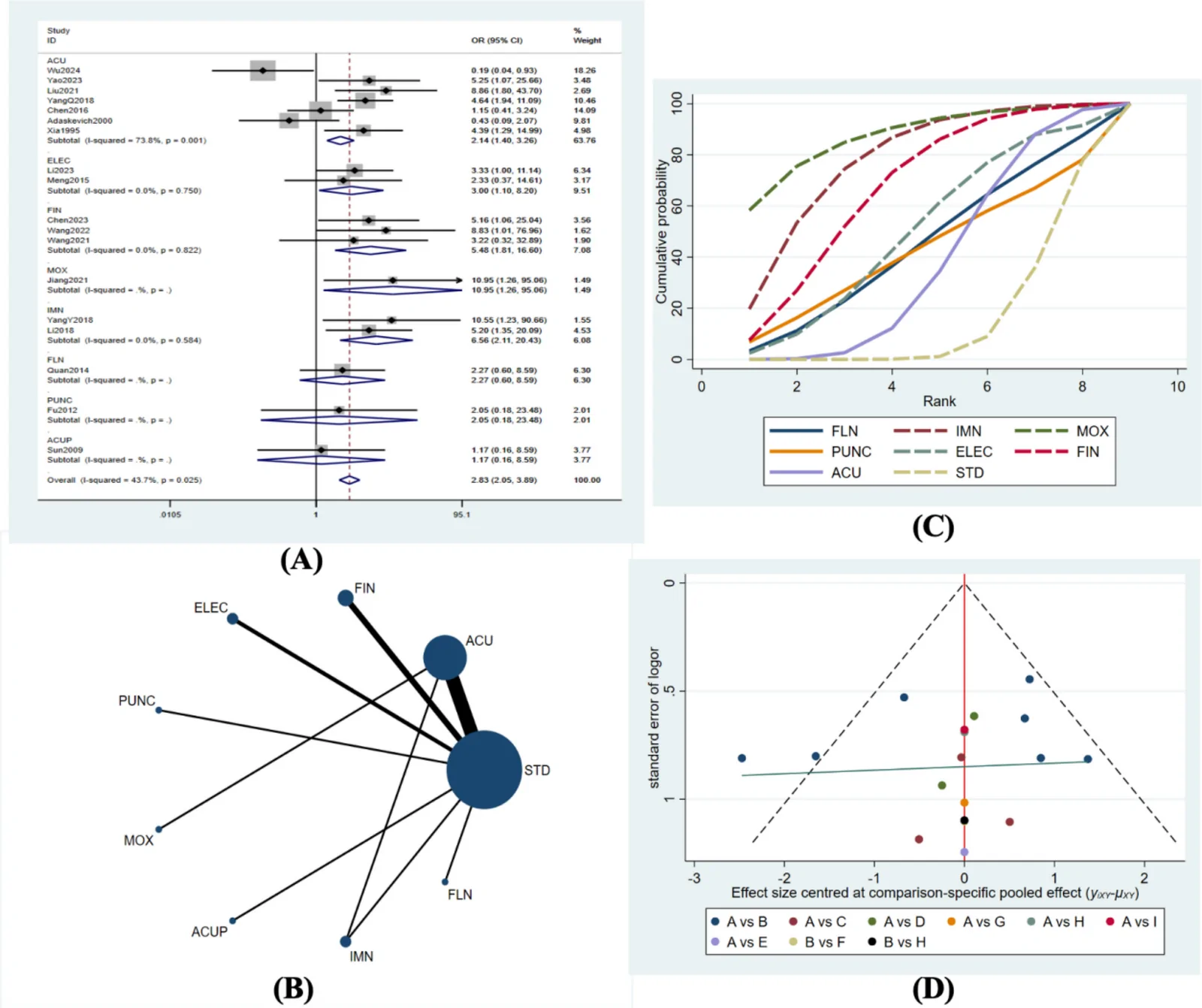
Different acupuncture and moxibustion therapies for ORR. (A) Forest diagram of the ORR; (B) network diagram of the ORR; (C) cumulative probability ranking plot of the ORR. (D) Funnel plot of the ORR. STD: standard care, ACU: acupuncture, FIN: fire needle, ELEC: electroacupuncture, PUNC: puncture and cupping, MOX: moxibustion, ACUP: acupoint application, IMN: embedding needle and FLN: flying needle

A total of 18 studies [17, 19–23, 25, 26, 28–30, 32–34, 36–39] used the overall response rate (ORR) as protocol-defined endpoints, involving 8 different interventions (including ACU, FIN, ELEC, PUNC, MOX, ACUP, IMN, and FLN). In the results of the traditional pairwise meta-analysis of ORR, Odds Ratios (ORs) were utilized as the primary effect size. An OR greater than 1 signifies that the experimental intervention has a higher probability of achieving a clinical response compared to the control group. The results demonstrated that acupuncture and moxibustion therapies significantly improved ORR in the treatment of AD patients compared with the control group (OR: 2.83, 95% CI: 2.05–3.89; I^2^ = 43.7%, P = 0.025). Specifically, ACU (OR: 2.14, 95% CI: 1.40–3.26), ELEC (OR: 3.00, 95% CI: 1.10–8.20), FIN (OR: 5.48, 95% CI: 1.81–16.60), MOX (OR: 10.95, 95% CI: 1.26–95.06), and IMN (OR: 6.56, 95% CI: 2.11–20.43) all demonstrated statistically significant improvements (Fig. 3A).

The network relationship between the interventions is shown in Fig. 3B. There was a closed loop between different interventions, and a node-splitting analysis was performed to assess consistency. The results revealed no statistically significant difference (Ratio of Odds Ratios (ROR) = 3.997, 95% CI: 1.00–331.95, P = 0.539), indicating good consistency. The consistency model (AIC = 81.144) and the inconsistency model (AIC = 79.49) showed comparable fit, with an AIC difference of less than 2. Combined with the results of the design-by-treatment interaction test (P = 0.539) and loop-specific tests (ROR = 3.997, P = 0.539), no significant evidence of inconsistency was identified. Therefore, the consistency model was deemed appropriate and robust for the final data synthesis.. Network meta-analysis revealed that ACP (OR: 8.71, 95% CI: 1.49–51.04), ELEC (OR: 20.92, 95% CI: 1.11–371.94), and ACUP (OR: 5.32, 95% CI: 1.15–24.75) were significantly superior to FLN. The detailed results are shown in Table 2. For the rank probability of all interventions, the network map collectively analysed 9 interventions, including 8 acupuncture and moxibustion interventions and 1 control intervention (standard care, STD). The effects of the interventions were ranked as follows: MOX (87.2%) > IMN (78.0%) > FIN (67.1%) > ELEC (49.9%) > FLN (44.2%) > PUNC (42.5%) > ACU (37.5%) > ACUP (28.2%) > STD (15.5%).

**Table 2:** Network meta-analysis results of ORR and SCORAD

FLN	1.85(0.82,4.20)	**5.32(1.15,24.75)**	2.88(0.56,14.79)	2.05(0.10,41.59)	**20.29(1.11,371.94)**	1.17(0.08,16.78)	**8.71(1.49,51.04)**	\	2.27(0.25,20.73)
−0.08 (−0.51,0.35)	IMN	2.87(0.50,16.38)	1.55(0.25,9.67)	1.11(0.05,25.03)	10.95(0.67,178.38)	0.63(0.04,10.24	4.70(0.77,28.81)	\	1.23(0.12,12.95)
\	\	ACUP	0.54(0.06,5.10)	0.38(0.01,11.30)	3.81(0.14,102.22)	0.22(0.01,4.76)	1.64(0.16,17.02)	\	0.43(0.03,6.30)
**0.26 (0.05,0.46)**	0.33 (−0.04,0.71)	\	MOX	0.71(0.02,21.94)	7.06(0.25,198.57)	0.41(0.02,9.27)	3.03(0.27,33.75)	\	0.79(0.05,12.37)
**0.34(0.04,0.64)**	0.42 (−0.11,0.94)	\	0.08 (−0.28,0.45)	PUNC	9.91(0.15,651.43)	0.57(0.01,31.82)	4.25(0.13,139.61)	\	1.11(0.03,46.47)
0.09 (−0.41,0.60)	0.17 (−0.49,0.84)	\	−0.16 (−0.71,0.39)	−0.24 (−0.83,0.34)	ELEC	0.06(0.00,2.98)	0.43(0.02,11.97)	\	0.11(0.00,4.32)
0.13 (−0.29,0.55)	0.21 (−0.39,0.81)	\	−0.13 (−0.60,0.35)	−0.21 (−0.73,0.31)	0.04 (−0.62,0.69)	FIN	7.43(0.30,181.35)	\	1.94(0.06,61.64)
0.42 (−0.13,0.97)	0.50 (−0.13,1.13)	\	0.16 (−0.34,0.67)	0.08 (−0.54,0.71)	0.32 (−0.42,1.07)	0.29 (−0.40,0.98)	ACU	\	0.26(0.02,4.42)
0.28 (−0.79,1.35)	0.36 (−0.75,1.47)	\	0.02 (−1.02,1.07)	−0.06 (−1.17,1.05)	0.19 (−1.00,1.37)	0.15 (−1.00,1.30)	−0.14(−1.30,1.02)	SHAM	\
0.09 (−0.43,0.60)	0.16 (−0.50,0.83)	\	−0.17 (−0.72,0.38)	−0.25 (−0.84,0.34)	−0.01 (−0.73,0.71)	−0.04 (−0.71,0.62)	−0.33(−1.08,0.42)	−0.19 (−1.38,0.99)	STD

Note: the lower left matrix is the result of the SCORAD network meta-analysis, and the upper right matrix is the result of the ORR network meta-analysis. STD: standard care, SHAM: sham acupuncture, ACU: acupuncture, FIN: fire needle, ELEC: electroacupuncture, PUNC: puncture and cupping, MOX: moxibustion, ACUP: acupoint application, IMN: embedding needle and FLN: flying needle

The publication bias of the included studies was detected via comparation-correction funnel plots, which were essentially symmetric. Most of the scattered points were located at the upper part of the triangle, and some studies were outside the 95% CI, suggesting some bias (Fig. 3D).

#### SCORAD

SCORAD was used as an outcome measure in 15 studies, involving 7 AM interventions (including ACU, FIN, ELEC, PUNC, MOX, IMN, FLN) and 2 control interventions (STD and sham acupuncture, SHAM). The results of the traditional pairwise meta-analysis revealed that AM therapies significantly reduced SCORAD scores compared with standard care (SMD: −0.24, 95% CI: −0.37 to −0.11, I^2^ = 0.0%, P = 0.784).

**Fig. 4:**
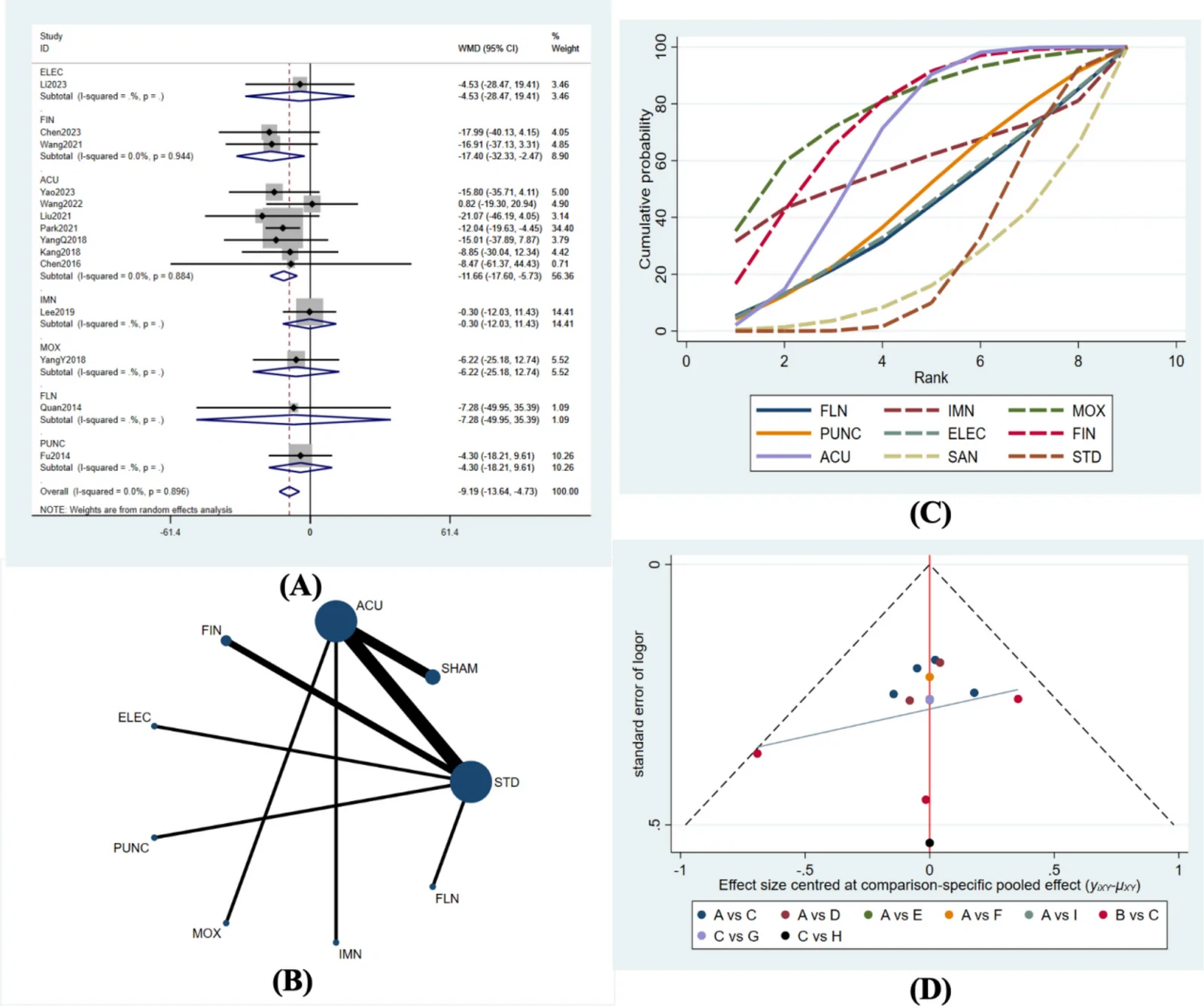
Different acupuncture and moxibustion therapies for SCORAD. (A) Forest diagram of SCORAD; (B) network diagram of SCORAD; (C) cumulative probability ranking plot of SCORAD. (D) Funnel plot of SCORAD. STD: standard care, SHAM: sham acupuncture, ACU: acupuncture, FIN: fire needle, ELEC: electroacupuncture, PUNC: puncture and cupping, MOX: moxibustion, IMN: embedding needle and FLN: flying needle

Owing to the absence of closed loops, the coexistence of both direct evidence and indirect evidence was not feasible. The results of the network meta-analysis demonstrated that, compared with MOX (SMD: 0.26, 95% CI: 0.05–0.46) and PUNC (SMD: 0.34, 95% CI: 0.04–0.64) (Table 2), FLN was significantly inferior in reducing SCORAD scores among AD patients. The rank probability analysis revealed the following hierarchical effectiveness ranking: MOX (77.9%) > FIN (74.1%) > ACU (64.8%) > IMN (58.0%) > PUNC (45.8%) > ELEC (41.9%) > FLN (41.1%) > STD (25.5%) > SHAM (20.8%) (Fig. 4C).

Publication bias assessment through adjusted funnel plots revealed fundamental symmetry, with most scattered points distributed along the central axis of the triangle, suggesting that there was no significant publication bias among the included studies (Fig. 4D).

#### Subgroup analysis by age group

The participants included in this study spanned a wide demographic range, from infants to adults. Considering that age-related differences in physiological characteristics and treatment tolerance may influence the clinical response to AM, we performed a dedicated subgroup analysis. In the 13 trials involving adults, AM therapies showed a significant advantage over controls (OR = 2.39, 95% CI: 1.67–3.44; I^2^ = 51.4%, P = 0.016). In the 5 trials involving children, the pooled effect was even more pronounced (OR = 5.05, 95% CI: 2.50–10.21) with zero heterogeneity (I^2^ = 0.0%). The consistent therapeutic direction across both subgroups reinforces the transitivity of the network and confirms that age did not fundamentally bias the superior performance of AM interventions.

#### Commonly used acupoints and time selection

Acupoint selection was predominantly from the limbs, with Xuehai (SP10) appearing 9 times (highest frequency). The remaining acupoints were as follows in descending order: Quchi (LI11, 8 times), Zusanli (ST36, 7 times), Sanyinjiao (SP6, 6 times), and Yinlingquan (SP9, 5 times). The treatment duration ranged from 3 to 12 weeks, with the 4-week cycle being the most common (n = 12). The frequency ranged from once to five times weekly, with three sessions per week being the most frequently administered (n = 10). For each session, 30 min was the most common duration (n = 11).

### Adverse events

Adverse events were reported in nine trials, with the most common being fatigue (1.1%), localized skin pruritus and pain (0.8%), and bleeding (0.6%). All reported adverse reactions were transient in nature, and no serious adverse events were documented.

## Discussion

### Summary of the main results

This study represents the first attempt to evaluate the efficacy and safety of various AM therapies in the management of AD, incorporating data from 23 studies involving a total of 1,658 patients. Given the diversity of acupuncture-based interventions, identifying the optimal therapeutic modality to maximize clinical efficacy remained the primary objective of our investigation. This study adopted both conventional pairwise meta-analysis and network meta-analysis approaches.

Our findings suggested that AM therapies were associated with significant improvements in the SCORAD index and ORR compared with those in the control group, which is consistent with the findings of a previous study [42]. Furthermore, our study provides further evidence by integrating direct and indirect comparisons, revealing that moxibustion (MOX), embedding needling (IMN), and fire needling (FIN) are among the most effective interventions. Among these, MOX was identified as the potentially most effective acupuncture-based therapy for improving symptoms of AD.

To evaluate the potential impact of age-related clinical heterogeneity, we performed a subgroup analysis. Our results demonstrated that while the magnitude of the effect may vary, the positive therapeutic trend remained remarkably stable across both adults and children. The higher OR observed in pediatric trials may be attributed to the specific AM modalities chosen for children (such as gentle moxibustion or acupoint application), which are often associated with high clinical responsiveness in acute-to-subacute presentations.

### Possible explanations for the present findings

From the perspective of traditional Chinese medicine (TCM), AD is predominantly attributed to disharmony of the lungs, spleen, and blood system, which is often characterized by wind, dampness, and heat pathogens invading the exterior and impairing the skin [43]. Moxibustion is traditionally believed to tonify Yang and dispel cold dampness, thereby enhancing defensive Qi (Wei Qi) and restoring internal balance. The preferential efficacy of MOX observed in our analysis aligns with this theoretical foundation, particularly for patients presenting with cold-type or deficiency syndromes [44].

Recent studies have highlighted the pivotal role of transient receptor potential vanilloid 1 (TRPV1) in the transmission of pruritic signals. TRPV1 functions as a downstream effector of both histamine receptors and nonhistaminergic protease-activated receptor-2 (PAR-2), contributing significantly to the pathogenesis of chronic itch. Notably, pharmacological inhibition of TRPV1 has been shown to alleviate scratching behavior in AD mouse models [45]. In line with these findings, mild moxibustion has been reported to suppress TRPV1 expression in the skin, thereby attenuating pruritus symptoms in murine models [46]. These results suggest that moxibustion may exert a targeted antipruritic effect through the modulation of TRPV1-mediated neuroinflammatory pathways, suggesting a plausible mechanism for its clinical efficacy in relieving intense itch in AD.

Similarly, the therapeutic superiority of IMN and FIN may be attributed to their distinct yet overlapping mechanisms of action, which target key immunological, neurological, and cutaneous pathways implicated in the pathogenesis of AD. FIN, which combines mechanical penetration with intense thermal stimulation, has been reported to enhance local microcirculation and facilitate dermal remodelling. The rapid application of heat may accelerate metabolic turnover and promote tissue regeneration [47]. IMN, a technique involving the subcutaneous implantation of absorbable threads at designated acupoints, provides continuous and prolonged stimulation. This sustained input is hypothesized to augment the regulation of neuroimmune interactions over time, potentially resulting in cumulative modulation of mast cell degranulation and localized cytokine release, thereby contributing to sustained symptom relief and a decreased frequency of disease flares [48].

### Selection of acupoints

An analysis of the included studies revealed that Xuehai (SP10) was the most frequently employed acupoint, appearing in 9 studies, followed by Quchi (LI11, 8 times), Zusanli (ST36, 7 times), Sanyinjiao (SP6, 6 times), and Yinlingquan (SP9, 5 times).

SP10, SP6, and SP9 are all Spleen Meridian and are frequently utilized to "regulate the blood division" (Tiao Xue Fen), a fundamental concept in treating disorders such as urticaria, eczema, and menstrual irregularities. SP10 (Xuehai) is particularly known for invigorating and cooling the blood, making it highly effective in cases involving blood heat-induced pruritus and chronic inflammatory skin diseases. LI11 (Quchi), situated on the Large Intestine Meridian, is traditionally used to "clear heat and expel wind" (Qing Re Qu Feng) and to "cool the blood and relieve itch" (Liang Xue Zhi Yang). Its frequent application in allergic dermatoses such as atopic dermatitis, urticaria, and acne vulgaris is consistent with its empirical role in resolving external heat and wind-pathogen invasions. ST36 (Zusanli), on the Stomach Meridian, is widely recognized for its immunoregulatory and tonifying effects, particularly in boosting the spleen and stomach Qi to "reinforce the upright Qi and dispel pathogenic factors" (Fu Zheng Qu Xie). When paired with SP10 and SP9, ST36 contributes to the harmonization of Qi and blood, which is central to the TCM pathophysiology of chronic skin conditions. SP6 (Sanyinjiao) harmonizes the liver, spleen, and kidney meridians and is often used in syndromes involving blood deficiency, dampness, or endocrine imbalance, which are common internal patterns in AD patients. SP9 (Yinlingquan), another Spleen Meridian point, facilitates water metabolism and dampness elimination and is frequently used to treat damp-heat skin lesions or edema when combined with SP10 and ST36 [49].

### Implications for clinical practice

To our knowledge, this is the first comprehensive network meta-analysis comparing multiple acupuncture strategies for atopic dermatitis, providing novel evidence that may inform future clinical guidelines. This study provides a foundation for the rational integration of acupuncture-based therapies into the clinical management of AD, highlighting the potential of nonpharmacological interventions to address chronic inflammation and pruritus. Beyond the comparative effectiveness of specific modalities, our findings underscore a broader need to align acupuncture treatment strategies with standardized syndrome differentiation and meridian-based acupoint selection, informed by both empirical practice and evidence-based guidelines.

For future clinical research, these results highlight several key directions. First, there is a critical need for rigorously designed, large-scale RCTs that directly compare high-ranking acupuncture modalities, such as moxibustion, fire needling, and embedding needling, with both conventional treatments and placebo controls. Second, stratified analyses on the basis of TCM syndrome patterns, disease severity, and age subgroups are essential to refine individualized treatment protocols. Third, studies should incorporate both short-term and long-term outcome measures, including recurrence rates and quality-of-life metrics, to better capture the holistic impact of acupuncture on AD. Furthermore, the integration of biomarkers, such as neuroimmune indicators, may help elucidate mechanistic pathways and bridge traditional concepts with contemporary biomedical understanding. Such research would not only strengthen the scientific credibility of acupuncture but also support its inclusion in multidisciplinary care models for chronic inflammatory skin diseases.

### Limitations

Despite the clinical relevance and potential translational value of our findings, several limitations must be acknowledged. First, a primary concern was the suboptimal methodological quality of the included studies. As noted during the peer-review process, ORR was a clinical endpoint highly susceptible to detection bias, particularly when participants and assessors were not blinded. Approximately 74% of the trials were conducted in East Asia, where implementing rigorous blinding in AM trials remains traditionally and practically challenging. Although our sensitivity analysis confirmed the robustness of the SCORAD results, the high proportion of "unclear" or "high" risk of bias in randomization and allocation concealment across multiple trials cannot be ignored. Consequently, we have downgraded the overall certainty of the evidence for both ORR and SCORAD. Therefore, moxibustion is characterized as a "promising" rather than "definitive" modality. Second, there was considerable heterogeneity in sample sizes and treatment durations across studies, potentially affecting the comparability and robustness of pooled efficacy estimates. Third, the majority of included comparisons were based on indirect evidence derived from network meta-analyses, with relatively few head-to-head RCTs directly comparing different acupuncture modalities. This reliance on indirect comparisons may reduce the overall confidence in treatment rankings and highlights the need for future RCTs that directly assess comparative effectiveness and safety. Fourth, the evaluation of publication bias via the funnel plot for ORR revealed potential small-study effects, as several studies fell outside the 95% confidence intervals. This is likely due to the predominance of small-scale, single-center Chinese trials and the inherent difficulty in implementing double-blinding for AM interventions. Such unblinded designs, particularly when measuring subjective outcomes like clinical response rates, may introduce performance and detection bias, potentially leading to exaggerated effect sizes. Furthermore, the availability of only a single RCT for certain interventions (e.g., acupoint application and puncture-cupping) further limits the generalizability of the findings. Therefore, while this study provides a relative ranking of AM modalities, the conclusions should be interpreted with caution, and the superior performance of specific techniques requires validation through larger, strictly blinded, multi-center trials.

## Conclusion

Compared with controls, acupuncture and moxibustion therapies significantly improved both SCORAD scores and overall response rates (ORRs). Among the interventions, moxibustion, embedding needling, and fire needling showed a higher probability of clinical efficacy according to SUCRA rankings. Nevertheless, the certainty of the evidence for many comparisons remains moderate to low, and several interventions were represented by only a single RCT. Therefore, while our study provides a valuable reference for clinical decision-making, practitioners should apply these findings with caution. Further high-quality, large-scale, multi-center RCTs are required to confirm the relative clinical superiority of specific AM techniques.

## Supplementary Information

Supplementary Material 1. Table S1. Definitions and evaluation criteria of Overall Response Rate (ORR) across included studies.

## Data Availability

Data sharing is not applicable to this article as no datasets were generated or analysed during the current study.
