# Supplementary material for: Different acupuncture and moxibustion therapies for atopic dermatitis: a systematic review and network meta-analysis
Source: Syst Rev. 2026 Sep 1;15:298. doi: 10.1186/s13643-026-03314-7 (PMC13769158; doi:10.1186/s13643-026-03314-7)
Supplement: Supplementary file 1 [file 13643_2026_3314_MOESM1_ESM.docx]

**Supplementary Table S1.** Definitions and evaluation criteria of Overall Response Rate (ORR) across included studies

| Study ID | Intervention | ORR Definition Criteria | Threshold for "Response" (Effective) |
| --- | --- | --- | --- |
| Wu 2024 | Acupoint injection | Clinical symptom and skin lesion reduction | SSRI≥30% (Cure + Excellence + Improvement) |
| Li 2023 | Electroacupuncture | SCORAD reduction index (SRI) | SRI≥20% (Recovery + Markedly effective + Improvement) |
| Chen 2023 | Fire needle | Composite index (SCORAD + EASI + DLQI) | Reduction≥25% (Cure + Markedly effective + Effective) |
| Yao 2023 | Acupuncture | Symptom Score Reduction Index (SSRI) | SSRI≥20% (Cure + Markedly effective + Effective) |
| Wang 2022 | Fire needle | Guiding Principles for Clinical Research of New TCM | SSRI≥50% (Cure + Markedly effective + Effective) |
| Wang 2021 | Fire needle | Guiding Principles for Clinical Research of New TCM | SSRI≥50% (Cure + Markedly effective + Improvement) |
| Liu 2021 | Acupuncture | SCORAD reduction rate | SSRI≥20% (Recovery + Markedly effective + Effective) |
| Jiang 2021 | Moxibustion | Score improvement rate | Improvement≥30% (Cure + Markedly effective + Effective) |
| Yang Q 2018 | Acupuncture | Guiding Principles for Clinical Research of New TCM | SSRI≥50% (Cure + Markedly effective + Improvement) |
| Yang Y 2018 | Embedding needle | SCORAD reduction rate | SSRI≥20% (Cure + Markedly effective + Improvement) |
| Li 2018 | Embedding needle | Clinical symptoms and signs reduction | Clinical improvement (Excellence + Effective) |
| Chen 2016 | Acupuncture | SCORAD reduction index | SSRI≥20% (Cure + Markedly effective + Effective) |
| Meng 2015 | Electroacupuncture | Symptom Score Reduction Index (SSRI) | SSRI≥20% (Cure + Markedly effective + Effective) |
| Quan 2014 | Flying needle | Curative effect index (Nimmo's Method) | SSRI≥20% (Recovery + Removal + Effective) |
| Fu 2012 | Puncture & Cupping | SCORAD reduction index | SSRI≥30% (Cure + Markedly effective + Effective) |
| Sun 2009 | Acupoint Stimulation | Skin lesion reduction rate | Reduction≥30% (Cure + Markedly effective + Effective) |
| Adaskevich 2000 | Acupuncture | Resolvement of clinical signs | Numerical improvement (Effective vs. Ineffective) |
| Xia 1995 | Acupoint injection | Clinical symptom resolution | SSRI≥65% (Cure + Markedly effective) |

Notes: SSRI = (Baseline Score-Post-treatment Score) / Baseline Score×100%.
